# A Non-Invasive Heart Rate Measurement Method Is Improved by Placing the Electrodes on the Ventral Side Rather Than the Dorsal in Loggerhead Turtles

**DOI:** 10.3389/fphys.2022.811947

**Published:** 2022-02-16

**Authors:** Chihiro Kinoshita, Ayaka Saito, Megumi Kawai, Katsufumi Sato, Kentaro Q. Sakamoto

**Affiliations:** ^1^International Coastal Research Center, Atmosphere and Ocean Research Institute, The University of Tokyo, Otsuchi, Japan; ^2^Atmosphere and Ocean Research Institute, The University of Tokyo, Kashiwa, Japan

**Keywords:** heart rate, sea turtle, non-invasive method, biologging, electrocardiogram

## Abstract

Heart rate measurement is an essential method for evaluating the physiological status of air-breathing diving animals. However, owing to technical difficulties, many marine animals require an invasive approach to record an electrocardiogram (ECG) in water, limiting the application of this approach in a wide range of marine animals. Recently, a non-invasive system was reported to measure the ECG of hard-shelled sea turtles by pasting the electrodes on the dorsal side of the shell, although the ECG obtained from the moving turtle contains noise produced by muscle contraction. Here, we report that clear ECGs can be obtained by placing the electrodes on the ventral side rather than the dorsal side in loggerhead sea turtles. Using our method, clearer ECG signals were obtained with less electrical noise, even when turtles are swimming. According to the anatomical features, the electrode position on the ventral side is closer to the heart than the dorsal side, minimizing the effects of noise generated by the skeletal muscle. This new biologging technique will elucidate the functioning of the circulatory system of sea turtles during swimming and their adaptabilities to marine environments. This article is part of the theme issue “Methods and Applications in Physio-logging.”

## Introduction

Diving animals, such as marine mammals, seabirds, and marine reptiles, are a group of breath-holding animals that have extended their habitats from land to sea. Diving animals change their oxygen consumption rate during diving (Castellini et al., 1992; Green et al., 2007; Ponganis, 2015). These animals show a variation in their heart rates over short time scales, such as diving bradycardia at the start of dive and tachycardia at the surface (Butler and Jones, 1997; Houser et al., 2010; Ponganis, 2015; Goldbogen et al., 2019; Williams et al., 2019; Okuyama et al., 2020; Sakamoto et al., 2021). They constrict peripheral blood vessels and decrease heart rates to maintain a constant blood pressure and distribute blood flow to only essential parts to ensure that oxygen-sensitive tissues remain adequately oxygenated under the water (Zapol et al., 1979; Schmidt-Nielsen, 1997; Jobsis et al., 2001). Thus, heart rate is an important indicator for understanding the cardiac response of diving animals (Ponganis, 2015). Previous studies have measured the electrocardiogram (ECG) and heart rate in various taxa of diving animals, especially in endothermic animals such as marine mammals and seabirds (Butler et al., 2004; Green, 2011).

Sea turtles, reptiles specialized in marine environments, are ectothermic diving animals. They have unique features among ectothermic diving animals because they perform deep (e.g., 1,186 m in leatherback turtles; 340 m in loggerhead turtles) (López-Mendilaharsu et al., 2009; Narazaki et al., 2015) and long (e.g., 614 min in loggerhead turtles; 330 min in green turtles) (Broderick et al., 2007; Fukuoka et al., 2015) dives and spend most of their lives at sea, except during nesting and hatching. Unlike endothermic animals, ectothermic animals such as sea turtles have low metabolic rates and hypoxic tolerance (Berkson, 1967; Lutz et al., 1980; Lutz and Bentley, 1985), and exhibit more modest reductions in heart rate during dives (Southwood et al., 1999; Williams et al., 2019; Sakamoto et al., 2021). Therefore, understanding the cardiac response of ectothermic diving animals will facilitate comparative studies on diving animals. However, owing to technical problems, studies on measuring ECGs and heart rates in diving animals have been biased toward endothermic diving animals and there is a lack of such knowledge in ectothermic diving animals (Green, 2011).

In Harms et al. (2007), alligator clips were attached to the skin and carapace of leatherback sea turtles on land to monitor their condition during anesthesia, and ECGs were measured by non-invasive approach. However, most studies measuring ECGs and heart rates in free-swimming loggerhead and green turtles have used invasive methods of inserting electrodes into the body (Southwood et al., 1999, 2003; Williams et al., 2019; Okuyama et al., 2020), because the bony carapaces of loggerhead and green turtles are believed to block the ECG signals. The surgical methods allow us to measure clear ECG and heartbeat signals of turtles in both laboratory as well as the field conditions; however, they are invasive and include incision or drilling to insert the electrodes into the body. These methods sometimes induce long handling stress, and it may take 2 weeks to heal the wound (Okuyama et al., 2020) owing to the animals’ low metabolic rates. Furthermore, the electrodes inserted into the body must be secured firmly to prevent the entry of seawater (Southwood et al., 2003; Williams et al., 2019). Sakamoto et al. (2021) developed a non-invasive method that allowed to obtain clear ECG signals by attaching electrode patches to the carapace while resting on land and in seawater. However, the ECG signals during the active phase, such as swimming, contain noise due to skeletal muscle constriction (Sakamoto et al., 2021). In humans, the attachment position of the electrodes is precisely defined to detect clear ECG signals. According to Brijs et al. (2018), the ECG signals in rainbow trout are stronger when the ECG logger is positioned close to the heart and comprises two electrodes facing the musculature in the ventral midline. In cetaceans, different attaching positions of the electrodes may result in less clear ECGs (Aoki et al., 2021). As the position of electrodes is one of the essential factors for measuring ECGs, the non-invasive method in sea turtles (Sakamoto et al., 2021) must be updated by re-examining the position of the electrodes to allow us to obtain clearer ECGs even during activity. If a non-invasive method could be developed to measure ECGs stably during the active phase, it would be an update over the available method that is applied widely with minimal stress for the animal. Furthermore, understanding the function of the circulatory system in sea turtles during their active phase would further our understanding of the adaptability of diving animals to the marine environment.

In this study, we updated the non-invasive method reported in Sakamoto et al. (2021) by changing the mounting position of the electrodes from the carapace (dorsal side) to the plastron (ventral side) to enable clear measurement of ECGs and heart rates in swimming sea turtles. We also compared the ECGs measured simultaneously on the dorsal and ventral sides and optimized steps for stable ECG measurements.

## Materials and Methods

### Animals and Study Site

Loggerhead turtles (*Caretta caretta*) were collected during June to August in 2021 from the Sanriku coastal area of the western North Pacific. These wild loggerhead turtles were accidentally captured in set nets in the Sanriku coastal area (38°17–39°28′ N, 141°24′–142°00′ E). Captured turtles were promptly transferred to tanks at the International Coastal Research Center (ICRC) of the Atmosphere and Ocean Research Institute, The University of Tokyo (39°21′05″ N, 141°56′04″ E). Following the definition of Bolten (1999), we measured the straight carapace length [*SCL* (cm)] from the notch to the tip of the carapace, as well as the body mass [*BM* (kg)]. The turtles were maintained for 5–55 days from the day of bycatch to the experiments. They were placed individually in an outside experimental water tank (155 × 115 × 60 cm deep) and fed Japanese common squid (*Todarodes pacificus*). The turtles were not fed during the ECG measurements. At the end of the experiments, all turtles were released into the sea near the ICRC.

### Electrocardiogram Logger and Behavioral Logger

The ECGs were recorded using an ECG logger (ECG400-DT; Little Leonardo, Tokyo, Japan; 21 mm width, 64 mm length, 23 mm height, 60 g mass in air). Lead wires for the electrode connections were extended from the ECG logger. The voltage span of the input signal was ± 5.8 mV. ECG signals were recorded at 250 Hz. We recorded data related to the acceleration (longitudinal axis) and depth using a behavioral logger (M190L-D2GT; Little Leonardo; 15 mm diameter, 53 mm length, 18 g mass in air). The acceleration was recorded at 16 Hz and the water temperature was recorded at 1 Hz.

### Step-by-Step Instructions for Attaching Electrocardiogram and Behavioral Loggers

**FIGURE 1:**
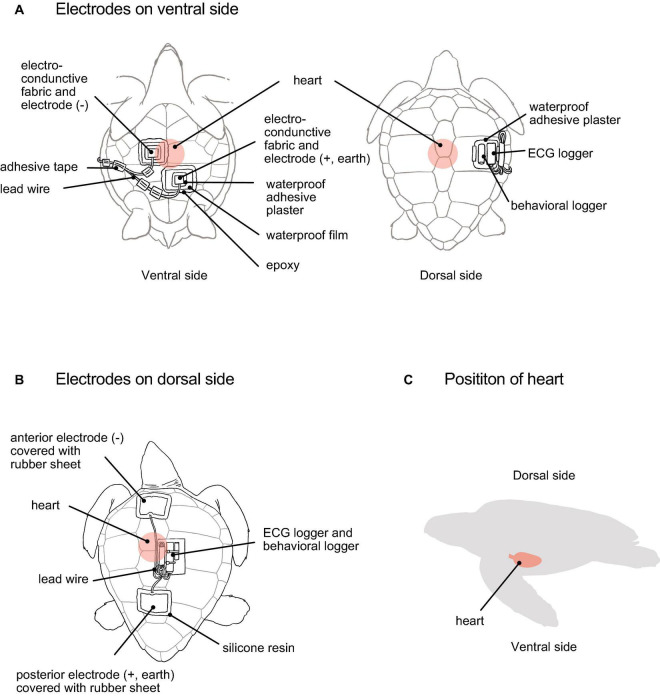
A loggerhead turtle with electrodes and ECG and behavioral loggers attached. The method of attaching electrodes on **(A)** ventral side (suggested by the present study) and **(B)** dorsal side (developed by Sakamoto et al., 2021). **(C)** A position of the turtle’s heart.

Sakamoto et al. (2021) recorded the ECGs of turtles using a non-invasive method that attached two electrode patches on the carapace of the turtle (dorsal side). In our study, electrodes were attached to the plastron (ventral side) and carapace, and ECGs were measured simultaneously to evaluate the effect of the attachment position (Figures 1A,B).

**FIGURE 2:**
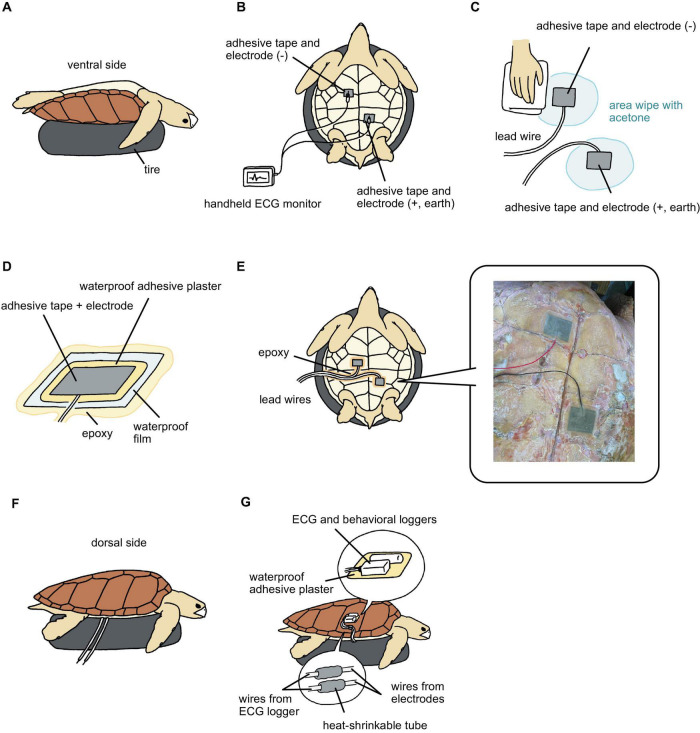
Step-by-step instruction of attachment the electrodes on the ventral side and the loggers on the dorsal side. **(A)** Place a tire underneath the turtles to hold them as upside down. **(B)** Two electrodes attached close to heart, and check ECG signals. **(C)** The plastron around the electrodes was wiped with acetone to avoid wetness. **(D)** Insulate the outside of the electrode with waterproof adhesive plasters and waterproof films. **(E)** Seal the edges of the waterproof film and lead wires with epoxy. **(F)** Turn over the turtle and placed on the tire. **(G)** Seal joint parts of lead wires with a heat-shrinkable tube. The loggers were fixed onto a waterproof adhesive plaster using an instant adhesive and then attached to the carapace.

For attaching the electrodes on the ventral side, we first placed a tire underneath the turtles to hold them (Figure 2A). At this time, the turtle was upside down. Adhesive tape made of electro-conductive material (KNZ-ST50 shield cloth tape; Kyowa Harmonet Ltd., Kyoto, Japan) was cut into rectangles (7 × 5 cm) and stuck to the lead wires for use as electrodes. Two electrodes were used to detect the voltage difference between the body parts. The anterior part was used as the negative electrode and located close to the heart, while the posterior part was used as both the positive and earth electrodes (Figure 2B). The advantages of using only two electrodes are that the minimum requirement ECGs can be obtained (not possible to measure detailed signals as 12 lead ECG in humans), fewer wires allow turtles to avoid entanglement, the handling time to attach electrodes can be shortened, and reducing the stress for animals. We looked for points where the amplitude of the R and T waves was the highest on carapace using a handheld ECG monitor (Checkme ECG ADV; SAN-EI MEDISYS Co., Ltd., Kyoto, Japan) (Figure 2B). After finding the most appropriate points, the electrodes were attached to the plastron. The electrodes were moistened with seawater to facilitate the detection of electrical signals. Then we checked again whether the electrodes detected the ECG signal using a handheld ECG monitor. Once the ECG signal was checked, the electrode edges were attached to the plastron using quick adhesive (Aron Alpha Jelly Extra; Konishi, Osaka, Japan). The plastron around the electrodes was wiped with acetone to avoid wetness (Figure 2C). Since the electrode was electroconductive, we insulated the outside of the electrode with waterproof adhesive plasters (Hydro Seal Extra Large; Johnson & Johnson, New Brunswick, NJ, United States) (7 × 5 cm) and waterproof films (FC Bosui Film; Hakujuji Co., Ltd., Tokyo, Japan) (10 × 10 cm) (Figure 2D). To complete the insulation between the plastron and the waterproof film, the edges of the waterproof films and lead wires were sealed with epoxy (Bond Quick 5; Konishi) (Figure 2E), which was allowed to cure for 5 min. After that, the turtle was carefully turned over and placed on the tire (Figure 2F). The two lead wires extending from the ECG logger were then connected to the lead wires from the electrodes. The joint part was sealed with a heat-shrinkable tube (Figure 2G). Then, the extra lead wires were covered with vinyl tape. We attached an ECG logger and a behavioral logger to the edge of the carapace (Figure 2G). The loggers were fixed onto a waterproof adhesive plaster using an instant adhesive and then attached to the carapace (dorsal side). After the loggers were attached, the turtles were left in the experimental water tank filled with seawater. This method required approximately 40 min.

We employed a technique previously described by Sakamoto et al. (2021) to attach the electrodes to the carapace (dorsal side) (Figure 1B). The two electrodes were placed on the midline anterior and posterior positions of the carapace and moistened with seawater. After ensuring that the ECG signals were detected properly, the edges of the electrodes were attached to the carapace using quick adhesive. At this point, the carapace around the electrodes was wiped with acetone, and the electrodes were covered with a rubber sheet (11 × 8 × 0.1 cm). The rubber sheet edges were sealed with silicone resin (Bath Bond Q; Konishi), which was allowed to stand for 30 min. The ECG logger and behavioral logger were attached to the center of the carapace (Figure 1B). After the loggers were attached, the turtles were left in the experimental tank filled with seawater. This method required approximately 60 min. After the experiments, all equipment were removed, and the data were downloaded.

### Checking Electrocardiograms and Estimating Heart Rates

The ECG logger detected signals (QRS complex: representing the depolarization of the ventricles) associated with turtle heartbeat. Following the methods of Sakamoto et al. (2021), the instantaneous heart rate was calculated as the reciprocal of the RR interval. The heart rate was estimated as the median instantaneous heart rate per minute. To process ECG signals, we used the ECGtoHR program (Sakamoto et al., 2021), which runs on IGOR Pro version 8.04 (Wavemetrics, Portland, OR, United States). When we use the program, initialization parameters were set as QRS frequency = 10 Hz, and Max heart rate = 60 bpm. ECG to HR removes noise from the ECG using a band-pass filter, detects R peaks, and estimates the heart rate per minute. The programs can be downloaded from the Internet.^1^ The ECG, water temperature, and acceleration data were analyzed using IGOR Pro with the Ethographer program package (Sakamoto et al., 2009).

To calculate the heart rates, the acceleration of the turtles was classified into “resting” and “active.” The recorded accelerations included low-frequency gravity components and high-frequency specific components (mainly caused by dynamic movements, such as flipper beating). The low- and high-frequency components were identified by a low-pass filter with different thresholds set for each individual (0.32, 0.43, and 0.40 Hz for Individuals L2105, L2109, and L2110, respectively). This filter was defined using for power spectral density plot and a continuous wavelet transform filter. We used the thresholds in order to separate the behavior (active and resting). After that, the standard deviation of the longitudinal acceleration was calculated every minute. If the standard deviation was less than 0.2 m s^–2^, the turtles were considered to be resting, because this value was very small and indicated no movements. The other phase was regarded as the active phase. IGOR Pro was used to analyze all acceleration data.

In this study, we prepared the two types of metrics that can quantify the increase in signal quality. First, the amplitudes were calculated from 100 randomly selected R waves in the original ECG (before filtering) on both dorsal and ventral sides and compared these data. Second, all R waves were clearly detected when measured on the ventral side, but some R waves were misidentified when measured on the dorsal side. The percentage of misidentification when measured on the dorsal side was calculated as the percentage of time when the estimated heart rate per minute differed by more than 10% from the value measured on the ventral side. Since an accurate measurement was made on the ventral side, it was considered that the heart rate could not be measured correctly on the dorsal side when the measured values were different on the dorsal side and the ventral side. All statistical analyses were conducted using R4.0.3 (R Development Core Team^2^). The significance level of statistical test set at *P* < 0.01. Mean ± SD values are presented unless otherwise indicated.

## Results

**FIGURE 3:**
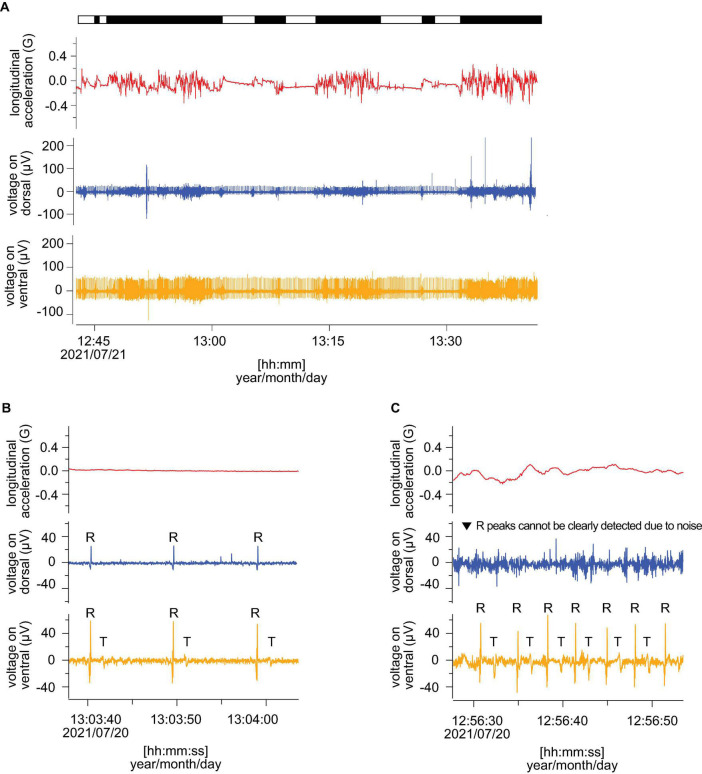
Comparison of the measured ECG (before filtering) from dorsal and ventral sides. Red, blue, and orange lines indicate longitudinal acceleration, ECGs from dorsal and ventral sides, respectively. **(A)** The example of ECGs including resting and active phases. The bar above indicated active (black) and resting (white) phases. **(B)** ECGs in resting and **(C)** active phase. The R and T indicate R peaks and T peaks. All panels show the data from L2110.

The ECGs and behavioral data were measured for three loggerhead turtles. The *SCL* and *BM* of turtles ranged between 68.5 and 79.5 cm and 53.5–78.5 kg, respectively (Table 1). The average water temperature during the experiments was 22.1, 19.3, and 20.5°C for L2105, L2109, and L2110, respectively. The recording durations were 20–23 h. The proportion of resting and active time were 24 and 76% for L2105, 38 and 62% for L2109, and 32 and 68% for L2110. Clear ECG and heartbeat signals were obtained from all individuals. Figure 3A indicates an example from an individual (L2110). For the original ECGs (before filtering), clear R peaks (positive oscillations in the QRS wave) were detected during the rest phase on the dorsal and ventral sides, but T peaks (oscillations caused by the repolarization of the ventricles) were only detected on the ventral side (Figure 3B). During the active phase, the amplitude of the R peaks was higher on the ventral side, and the heartbeats were clear even if there was noise (Figure 3C). However, when measured from the dorsal side, some R peaks in the active phase were offset by the noise due to muscle contractions as the amplitudes of these peaks were small (Figure 3C). The T peaks during the active phase were detected only on the ventral side (Figure 3C). The averaged amplitudes of R peaks were 64.5 ± 3.4, 67.3 ± 2.1, 91.9 ± 9.5 μV on the ventral side in L2105, L2109, and L2110, respectively. On the other hand, these were 43.3 ± 0.5, 28.8 ± 3.4, 35.8 ± 10.9 μV on the ventral side in L2105, L2109, and L2110, respectively. The amplitudes were 1.5–2.6 times higher on the ventral side than the dorsal side in each individual, and it is indicated that improvement of accuracy (Wilcoxon rank-sum test; *W* = 8,100, *P* < 0.001 for L2105, *W* = 6,570, *P* < 0.001 for L2109, *W* = 3,340, *P* < 0.001 for L2110).

**TABLE 1:** Individual data, recording duration, and heart rates as means ± SD.

Turtle ID	*SCL* (cm)	*BM* (kg)	Record durations (h)	Time ratio (%)		Heart rate from ventral side (beats min^–1^)		Heart rate from dorsal side (beats min^–1^)	
			
				Resting	Active	Resting	Active	Resting	Active
L2105	79.5	78.5	21	24	76	5.2 ± 1.7	15.3 ± 5.7	5.2 ± 1.5	13.5 ± 6.4
L2109	68.5	53.5	20	38	62	4.2 ± 0.7	11.3 ± 4.5	4.4 ± 1.5	22.3 ± 8.1
L2110	71.0	57.0	23	32	68	4.7 ± 1.9	11.6 ± 5.3	5.2 ± 3.6	14.4 ± 7.5

**FIGURE 4:**
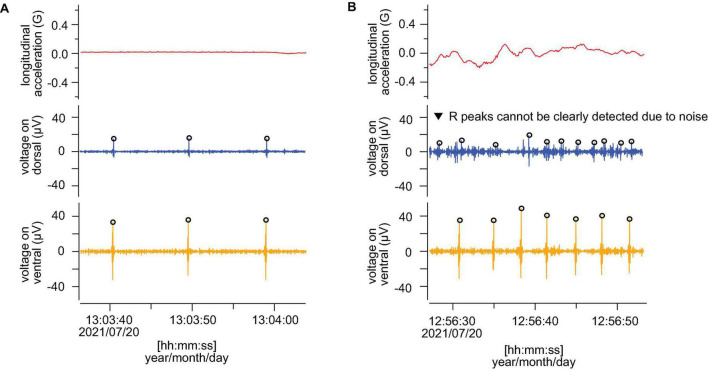
Comparison of the measured ECG (after filtering) from dorsal and ventral sides. **(A,B)** Show resting phase and active phase, respectively. Red, blue, and orange lines indicate longitudinal acceleration, ECG from dorsal and ventral sides, respectively. The open circles show the points judged as R wave by the program in Sakamoto et al. (2021). All panels show the data from L2110.

By comparing the ECGs after removing noise using the program developed by Sakamoto et al. (2021), the R peaks in the resting phases were clearly detected on both the dorsal and ventral sides (Figure 4A). In contrast, during active phases, the R peaks were more clearly detected on the ventral side than the dorsal side in all individuals (Figure 4B). The heart rates could not be counted properly on the dorsal side due to strong noise in some active phases (Figure 4B). On the ventral side, the averaged resting heart rates were 5.2 ± 1.7, 4.2 ± 0.7, and 4.7 ± 1.9 beats min^–1^ for L2105, L2109, and L2110, respectively, and averaged active heart rates were 15.3 ± 5.7, 11.3 ± 4.5, and 11.6 ± 5.3 beats min^–1^ for L2105, L2109, and L2110, respectively (Table 1). On the dorsal side, the averaged resting heart rates were 5.2 ± 1.5, 4.4 ± 1.5, and 5.2 ± 3.6 beats min^–1^ for L2105, L2109, and L2110, respectively, and averaged active heart rates were 13.5 ± 6.4, 22.3 ± 8.1, and 14.4 ± 7.5 beats min^–1^ for L2105, L2109, and L2110, respectively (Table 1). The percentage of misidentification when measured on the dorsal side was 1.3, 19.5, and 16.4% (resting) and 42.8, 87.2, and 51.0% (active) for L2105, L2109, and L2110, respectively. The accuracy of detected heart rates during activity was more improved.

## Discussion

In this study, we optimized a step-by-step non-invasive method to detect the clear ECG signals of sea turtles and compared the ECG and heartbeat signals measured simultaneously on the ventral and dorsal sides of turtles. The updated method of attaching the electrodes on the ventral side allowed us to measure the ECG and heartbeat signals clearly (without strong noises) during both the resting and active phases. Furthermore, when the electrodes were attached to the ventral side, the T peaks were clearly detected in both active and resting phases.

The ECG signals were more clearly detected because the ventral side is closer to the heart than the dorsal side (Figure 1C). Furthermore, the myogenic artifacts were smaller on the ventral side than on the back (Figure 3C), even though the ventral side was also closer to the pectoral muscles. We could not elucidate the causes of small myogenic artifacts on ventral side. The intensity of the ECG signals varies according to the position of electrodes in fish and cetaceans (Brijs et al., 2018; Aoki et al., 2021), and for the same reason, the position of electrodes is important in loggerhead turtles. In this study, a negative (anterior) electrode was placed to the right of the heart, i.e., between the humeral and pectoral scutes, and a positive (posterior) electrode was placed to the left of the heart, i.e., between the abdominal and femoral scutes on the plastron (Figure 1A). If the electrodes were displaced from this position or attached to places other than the plastron, the ECG signals became weak or undetectable. It was also important that the moist electrodes were closely attached to the plastron surface, but were sealed to prevent the sea water from entering. The quality of the materials of the plastron and the distance to the lungs, which contain air and less electrically conductive, should also be relevant and needs to be evaluated.

However, for using this updated method, one must be careful to ensure that the patches do not rub against the substrate and fall off. Thus, this method should be suitable for both resting and active turtles under water, but not for turtles at nesting grounds, as they tend to rub their ventral sides. Moreover, although this method is appropriate for measuring the ECGs of loggerhead turtles, the applicability to other sea turtle species has not been tested. According to Sakamoto et al. (2021), ECGs and heart rate measurements were difficult in green turtles. During the preliminary experiments of our study, we attempted to measure the ECGs of green turtles from not only carapace, but also limbs, neck, head, and armpits by using a handheld ECG monitor, but were also unable to detect clear ECGs. In Sakamoto et al. (2021), clear ECGs of black turtles, closely related to the green turtles, were measured on the dorsal side. Thus, the problem above might not be due to cardiac depolarization vector but to species-specific scute texture or body composition in green turtles. In our study, as the accuracy of ECG signals increased, the detectability of T peaks, which are waves produced by repolarization of the ventricle, also increased. The T waves represent the repolarization of the ventricles. Thus, by detecting both R and T waves, it is possible to gain deep insight into arrhythmias. It has been reported that arrhythmia may occur when air-breathing animals dive deeply (Williams et al., 2015). These types of arrhythmias are only observed in the deep sea and require further research for ectothermic animals.

This would enable us to understand heartbeats in detail, such as the excitation and impulse transmission intervals. Our non-invasive method is less damaging to sea turtles and can be used for health management for rescued individuals. Our method should also allow us to provide clearer ECG and heartbeat signals of loggerhead turtles during swimming in field conditions. For example, combined with an automatic time-scheduled release system as reported by Watanabe et al. (2004) and Narazaki et al. (2009) the ECGs could be measured for several days with minimal stress. The comprehensive ECG and heart rate measurements under the specific events that only occur in the field condition, especially deep dive, will contribute to elucidating the mechanism of cardiac adjustment in ectothermic diving animals and their adaptation to the marine environment.

## Data Availability Statement

The original contributions presented in study are included in the article/Supplementary Material, further inquiries can be directed to the corresponding author.

## Ethics Statement

The animal study was reviewed and approved by the Animal Ethics Committee of Atmosphere and Ocean Research Institute, The University of Tokyo.

## Author Contributions

CK and AS conceived the study and analyzed the data. CK, AS, KS, and KQS designed the experiment. CK, AS, MK, and KS conducted the experiment. CK wrote the first draft. All authors revised the manuscript and approved the final version.

## Conflict of Interest

The authors declare that the research was conducted in the absence of any commercial or financial relationships that could be construed as a potential conflict of interest.

## Publisher’s Note

All claims expressed in this article are solely those of the authors and do not necessarily represent those of their affiliated organizations, or those of the publisher, the editors and the reviewers. Any product that may be evaluated in this article, or claim that may be made by its manufacturer, is not guaranteed or endorsed by the publisher.

## Footnotes

1
https://sites.google.com/site/kqsakamoto/ecgtohr

2
http://www.R-project.org
